# Different Parts of the Chicken Embryo Egg Improve D-Galactose-Induced Aging in a Mice Model

**DOI:** 10.1155/2021/6654683

**Published:** 2021-04-27

**Authors:** Hsin-Tai Hong, Tsung-Hsien Hsu, Shao-Wen Hung, Chien-Chao Chiu, Chun-Yun Wu, Chia-Chi Chen, Guo-Chi Lee, Jyh-Shiun Lin, Chean-Ping Wu

**Affiliations:** ^1^Department of Animal Science, National Chiayi University, Chiayi 600, Taiwan; ^2^Division of Animal Industry, Animal Technology Research Center, Agricultural Technology Research Institute, Miaoli 350, Taiwan; ^3^Department of Science and Technology, Council of Agriculture Executive Yuan, Taipei 100, Taiwan

## Abstract

Chick (CE) or duck embryo eggs are known for nutritional supplement foods in traditional East countries for physical fitness enhancement and postpartum conditioning for many years. In this study, we evaluated the effects of different parts of the 10-day CE (embryo: CEr, yolk: CEw, and chorioallantoic membrane: CEp) on the antifatigue and antiaging activities in a D-galactose- (D-gal) induced aging mice model. The results showed CEp obviously increased the muscle weight and the liver and muscle glycogen content and enhanced exercise performance. In the antiaging assay, CEp significantly increased the activity of superoxide dismutase (SOD) and Glutathione Peroxidase (GPx). Moreover, the immunohistochemistry results of NRF-2 and HO-1 were also detected in the livers of mice in the D-gal/CEp group. The only partially potential such as CEr might improve OFT function with TG level, and CEw had strange grip strength. Therefore, we suggest that CEp has a potent antifatigue ability and could minimize the occurrence of age-associated disorders, more than other parts of the 10 days chicken embryo egg.

## 1. Introduction

Aging is associated with a progressive physiopathological process that results in health complications and diseases, including neurodegenerative diseases, cardiovascular diseases, and cancer [1]. The resulting problems of the global aging population increase have become a huge social burden. Reversing aging has become an important part of biomedical research [2]. Animal aging models are important for aging studies, and several standard models have been established, including fruit flies, fish, birds, mice, rats, and dogs [3, 4]. There are two types of research on aging animal models: the naturally aging model and the accelerated aging models [5, 6]. However, accelerated aging models are widely and commonly used [5]. D-Galactose (D-gal) is a chemical compound that has been used as an accelerated aging model in mice and rats [7, 8]. D-Gal contributes to the generation of reactive oxygen species (ROS) via the D-gal metabolism [9]. Ho et al. research showed that D-gal-induced aging mice exhibit progressive declines in learning and memory ability, mobility, and cardiac dysfunction, which are similar to those observed in naturally aging mice [10].

Many studies have shown that D-gal induces accelerated aging, including atrophy of skeletal muscle with a significantly reduced muscle mass/body mass ratio, cross-sectional area, and fiber diameter of skeletal muscle in D-gal-induced aging rats, when compared to normal control rats [11]. Wei and colleagues showed that D-gal can induce behavioral impairment in mice [12]. In addition, reactive oxygen species (ROS) play an important role in aging because ROS can disturb the homeostasis of cells and tissues, which ultimately threatens the integrity of the organism [13, 14]. Rodents chronically injected with D-gal have been used as an animal aging model.

In the food and pharmaceutical industries, there has been considerable interest in the search for natural materials to inhibit free radical production, maintain physical fitness, and postpone the aging process. The chick or duck embryo eggs are considered nutrition supplements for the elderly and pregnant women in China and Vietnam [15]. A previous study reported that chick embryo eggs (CE) have abundant nutritional mixtures including various amino acids, vitamins, phospholipids, and growth factors [16, 17]. In recent studies, the bioactive functions of embryo chicken egg have been explored to exhibit antioxidation and antifatigue effects in vivo. However, the bioactive functions of different parts of CE and the related mechanism are limited. In this study, a D-gal-induced aging mouse model was used to evaluate the effect of different parts of CE against aging induction. The physiological fatigue, antioxidant enzyme activity, plasma biochemical parameters, tissue glycogen content, and histologic changes were investigated. Taken together, we evaluated the potential aging prevention and antifatigue effects of different parts of CE supplementation in a D-gal-induced aging mouse model.

## 2. Materials and Methods

### 2.1. Preparation of Different Parts of a 10-Day Chicken Embryo Egg

Embryo Leghorn chicken eggs were obtained from the Animal Drugs Inspection Branch of the Animal Health Research Institute. Teen-day-old embryo chicken egg samples were obtained by incubating in the laboratory incubator for 10 days. The eggs were incubated at 37.8°C with a relative humidity of 60%-63% as reported previously. The different parts of embryo (CEr), yolk (CEw), and chorioallantoic membrane (CEp) samples were then minced, freeze-dried, pulverized to powdery granules, and stored at −20°C. The nutritional ingredients of the CE parts were analyzed by SGS Taiwan Ltd. (SGS Taiwan limited, New Taipei, Taiwan) and shown in Table 1. Normal saline was used to prepare the samples into CEr, CEw, and CEp suspensions before use.

### 2.2. D-Gal-Induced Aging Animal Model and Treatment

Male C57BL/6 mice (7-8 weeks old, weighing 20 ± 1.6 g) were purchased from BioLASCO Corp (Yilan, Taiwan). The animals were housed under the following conditions: 23 ± 2°C with a relative humidity of 60 ± 10%. They were fed a standard diet and water *ad libitum* throughout the experimental period. All experiments were conducted in accordance with the guidelines of the Institutional Animal Care and Use Committee (IACUC) of the Agricultural Technology Research Institute. This study was approved by the IACUC ethics committee, under protocol 109025.

Six to seven mice were randomly assigned to each of four groups: D-gal group (D-gal treated only), D-gal/CEr, D-gal/CEw, and D-gal/CEp groups. The control group was subcutaneously injected with the same volume of physiological saline. All groups were injected intraperitoneally with D-gal (200 mg/kg, Sigma, St Louis, MO, USA) once daily, for 120 days. Meanwhile, D-gal plus different embryonic extracts of CEr, CEw, or Cep were oral gavage administered for 120 days. In addition, 10-11 week-old mice (6 mice in this group) were used as a young control group. At the end of the experiments, all mice were killed via asphyxiation using 95% CO_2_, and the serum, organs, and tissues of mice were immediately collected for different assays and analyses.

### 2.3. Forelimb Grip Strength, Four-Limb Hang Time, Exhaustive Swimming Exercise, and Rotarod Test

A grip strength meter (Model-RX-5, Aikoh Engineering, Nagoya, Japan) was used to measure the grip power of the forelimb. The tensile force in each mouse was measured using a force transducer equipped with a metal bar (2 mm in diameter and 7.5 cm in length). We grasped the mouse at the base of the tail and lowered it vertically toward the bar. Once the two paws (forelimbs) grasped the bar, the mouse was pulled slightly backward by the tail, which triggered a “counterpull.” The grasping force was recorded by a grip strength meter in grams [18]. Forelimb grip strength was tested at 106 days of experiment beginning.

For the four-limb hanging test, position the grid 25 cm above soft bedding not only to prevent mice from harming themselves upon falling but also to discourage mice to intentionally jump off the grid. Place the mouse on the grid so that it grasps it with its four paws and start the timer. The test ended after two sessions. The maximum hanging time was used for the analysis [19]. Four-limb hanging was tested at 108 days of experiment beginning.

The rotarod test protocol was slightly modified on previous report [20]. Mice were placed on the rotarod (755 Rotarod; IITC Life Sciences, Wool and Hills, GA, USA) at a speed of 5 rpm, which was gradually accelerated to 10 rpm. The mouse stays time of on the rotating rod for analysis. This test was tested at 110 days of experiment beginning.

For a swim-to-exhaustion test, a weight that was equivalent to 5% of their body weight was attached to their tails. The mice were individually placed in a columnar swimming pool (height, 30 cm and radius, 10 cm) with a 20 cm water depth, maintained at 27°C ± 1°C. The swimming exhaustion of mice was reached when there was the loss of coordinated movements and failure to return to the surface within 7 s [18, 21]. This challenge was perform at 113 days of experiment beginning.

### 2.4. Open Field Test

The locomotor activity of mice was measured in a 50 cm × 50 cm open field arena (50 cm × 50 cm × 38 cm, length × width × height). The mice were placed in the center of the apparatus, and their locomotor behaviors were recorded for 10 min using a digital camera. The camera was connected to a computer, and ANY-maze software (San Diego Instruments) was used to track and analyze the movement in real time. The horizontal locomotor activity was expressed as the total ambulatory distance. The open field chamber was cleaned between trials using a 75% alcohol solution.

### 2.5. Liver and Muscle Glycogen Determination

The liver and muscle tissues were weighed prior to glycogen content analysis. A total of 100 mg of liver or muscle tissues was homogenized in 0.5 mL of cold 10% perchloric acid. After centrifugation at 15,000 × *g* at 4°C for 15 min, the supernatant was carefully recovered and kept on ice prior to analysis. Tissue extracts (30 *μ*L) were added to 96-well microplates, and 200 *μ*L of iodine-potassium iodide reagent was added to each well. An amber-brown compound developed immediately after the reaction. After allowing the plate to rest for 10 min, an ELISA reader (Thermo Multiskan GO, Thermo Fisher Scientific, Vantaa, Finland) was used to measure the absorbance.

### 2.6. Serum Chemistry Assessments

The serum levels of alanine aminotransferase (ALT), aspartate aminotransferase (AST), blood urea nitrogen (BUN), triglyceride (TG), lactate dehydrogenase (LDH), creatine kinase (CK), and glucose were assessed using a clinical chemistry autoanalyzer (Hitachi 7060, Hitachi, Tokyo, Japan).

### 2.7. Tissue Glycogen Determination

The liver and skeletal muscles are the two major glycogen deposition sites. Hence, we investigated whether the glycogen content in these two target tissues was influenced by different parts of the CE in the D-gal aging model. The glycogen content of the liver and muscle tissues was analyzed as previously described [18].

### 2.8. Catalase (CAT), Superoxide Dismutase (SOD), and Glutathione Peroxidase (GPx) Activity

The liver and muscle samples were homogenized with phosphate-buffered saline (PBS) and then centrifuged at 16,000 × *g* for 10 min at 4°C. The supernatant was quantified using CAT, SOD, and GPx assay kits, according to the manufacturer's instructions. The Catalase Assay Kit (Cayman, MI, USA) was used to evaluate the activity of CAT via the decomposition of hydrogen peroxide. A GPx assay kit (Cayman, MI, USA) was used to determine the activity of GPx, which converted reduced glutathione into oxidized glutathione utilizing GPx and oxidized NADPH into NADP^+^.

### 2.9. Histopathological Examination

The liver, kidneys, epididymal fat, spleen, pancreas, heart and muscles (gastrocnemius and soleus) fixed in 10% neutral-buffered formalin for 1 day, dehydrated, embedded in paraffin, sectioned into 4 *μ*m slices, and stained with hematoxylin and eosin (H&E) for histological examination.

### 2.10. Immunohistochemical Staining (IHC)

Liver sections (4 *μ*m thick) were deparaffinized in xylene, rehydrated using graded concentrations of ethanol, immersed in 3% hydrogen peroxide to block the endogenous peroxidase, and washed in phosphate-buffered saline. Tissue sections were heated in 10 mmol/L sodium citrate (pH 6.0) in a water bath at 95°C for 10 min to expose the tissue's antigens and then incubated at 4°C overnight with diluted (1 : 200) mouse monoclonal anti-heme oxygenase-1 (HO-1) antibody or rabbit polyclonal antinuclear factor erythroid-2-related factor 2 (NRF-2) antibody (Novus Biologicals LLC, Centennial, CO). Sections were then washed and incubated with Picture™ HRP polymer conjugate (Invitrogen, South San Francisco, CA) at room temperature, for 20 min. After washing, HRP localization was visualized using Dako AEC reagent (Zymed, South San Francisco, CA). The number of HO-1 and NRF-2 positive cells were determined in 400x microscopic fields by veterinarian (Dr. Chien-Chao Chiu).

### 2.11. Statistical Analysis

Data are presented as the mean ± standard error of the standard deviation (SD). Statistical analyses were performed using GraphPad Prism version 7 (GraphPad Software, Inc., La Jolla, CA, USA). Differences between groups were analyzed using one-way ANOVA. A *P* value of less than 0.05 was considered significant (^✽^) and less than 0.01 as highly significant (^✽✽^) when compared with the D-gal group.

## 3. Results

### 3.1. Effects of Supplementation with Different CE Parts on D-Gal-Induced Body Weight and Organ Weight Change

**Figure 1:**
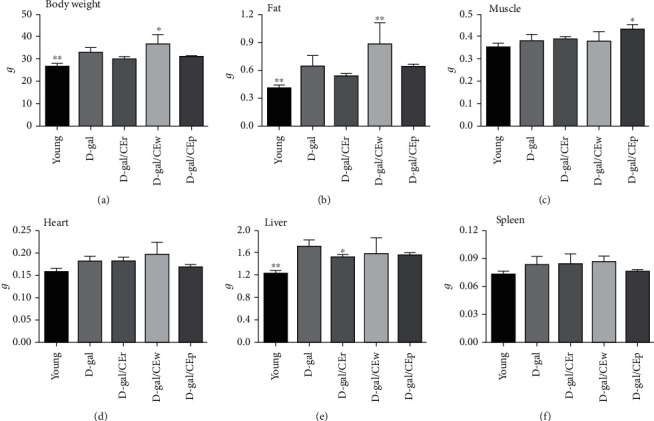
Effects of different parts of CEs on D-gal-induced aging model. (a) Body weight, (b) fat weight, (c) muscle weight, (d) heart weight, (e) liver weight, and (f) spleen weight. Data are expressed as means ± standard deviation (SD).^∗^*P* < 0.05 or ^∗∗^*P* < 0.01 compared with the D-gal group.

There were no significant differences in the initial body weights in each group. After D-gal challenge, the body weight was slightly higher in the D-gal group than in the young group. A significant increase in body weight was observed in the D-gal/CEw group, compared with the D-gal group. No differences were observed in the other groups (Figure 1, Figure S1). As shown in Figure 1, the epididymal fat weight was significantly higher in the D-gal/CEw group than in the D-gal group. There was a marked increase in the skeletal muscle (gastrocnemius and soleus muscles) weight in the D-gal/CEp group, compared with the D-gal group. In contrast, the liver weights were markedly lower in the young and D-gal/CEr groups than in the D-gal group. There were no significant differences in organ indices of the heart and spleen of mice between the groups.

### 3.2. Effect of Different CE Parts on the Forelimb Grip Strength, Four-Limb Hang Time, Rotarod, and Exhaustive Swimming Test

**Figure 2:**
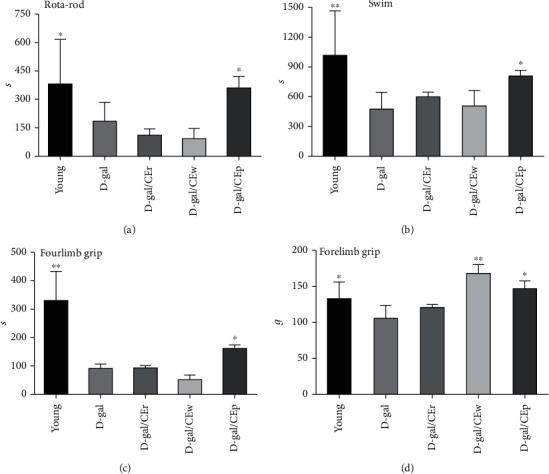
Effects of different parts of CEs on D-gal-induced fatigue during (a) rotarod test, (b) exhaustive swimming, (c) four-limb hang time, and (d) forelimb grip strength tests. Data are expressed as mean ± standard deviation (SD) for each group. ^∗^*P* < 0.05 or ^∗∗^*P* < 0.01 compared with the D-gal group.

The rotarod, grip strength, and exhaustive swimming test were performed to assess the effect of different CE parts on exercise performance. As shown in Figure 2, the rod residence time, exhaustive swimming time, and four-limb hang time of the D-gal/CEp group were 355 ± 171.1 s, 800 ± 181.2 s, and 157 ± 36.4 g, which were significantly longer than those of the D-gal group, which were 180 ± 103.3 s, 474 ± 171.1 s, and 89 ± 16.6 g, respectively (*P* < 0.05). The forelimb grip strength in the D-gal/CEp and D-gal/CEw groups was significantly increased in comparison to that of the D-gal group. Taken together, these experiments demonstrated that CEp was able to relieve fatigue.

### 3.3. Supplementation with Different CE Parts Preserves Spontaneous Activity, following D-Gal Treatment

**Figure 3:**
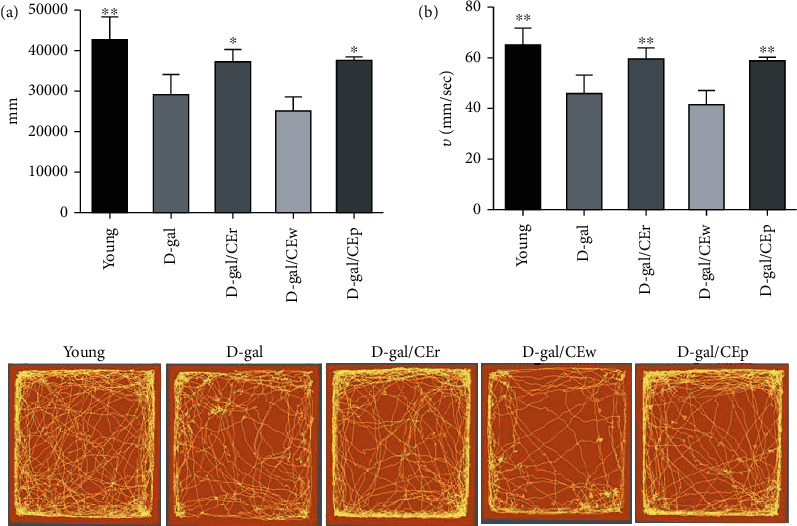
Effects of different parts of CEs on D-gal-induced alterations in locomotor activity in the open field test. (a) Total distance covered during the test. (b) Average velocity. ^∗^*P* < 0.05 or ^∗∗^*P* < 0.01 compared with the D-gal group.

Similar to previously published work, we found a large decrease in movement distance in D-gal mice, compared to young mice (Figure 3). Interestingly, compared with the D-gal group, the movement distance of mice was significantly increased in the D-gal/CEp and D-gal/CEr groups. The movement distances recorded were 37,256 ± 2,884 cm and 36,985 ± 8,634 in the D-gal/CEr and D-gal/CEp groups, respectively, and 28,957 ± 5,091 cm in D-gal group. In the case of average velocity, similar results were obtained.

### 3.4. Supplementation of Different CE Parts on Hepatic and Muscular Glycogen Levels via D-Gal Treatment

**Figure 4:**
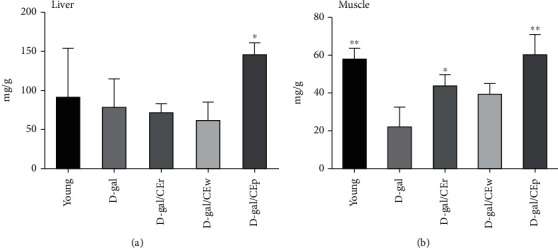
Effects of different parts of CEs on (a) hepatic and (b) muscular glycogen levels in D-gal-induced aging model. Data are expressed as means ± standard deviation (SD) in each group. ^∗^*P* < 0.05 or ^∗∗^*P* < 0.01 compared with the D-gal group.

Glycogen content is an integral determining factor in fatigue. In Figure 4, only the D-gal/CEp group showed a significantly elevated glycogen storage in the liver, compared to the D-gal group. Moreover, elevated muscle glycogen concentrations were found in the D-gal/Cep (60 ± 23.5 mg/g), D-gal/CEr (43 ± 14.2 mg/g), and young (57 ± 6.4 mg/g) groups, compared to the D-gal (21 ± 11.0 mg/g) group. There were no differences between the D-gal/CEw and D-gal/CEr groups.

### 3.5. Effect of the Supplementation Using Different CE Parts on Biochemical Analyses at the End of the Experiment

**Figure 5:**
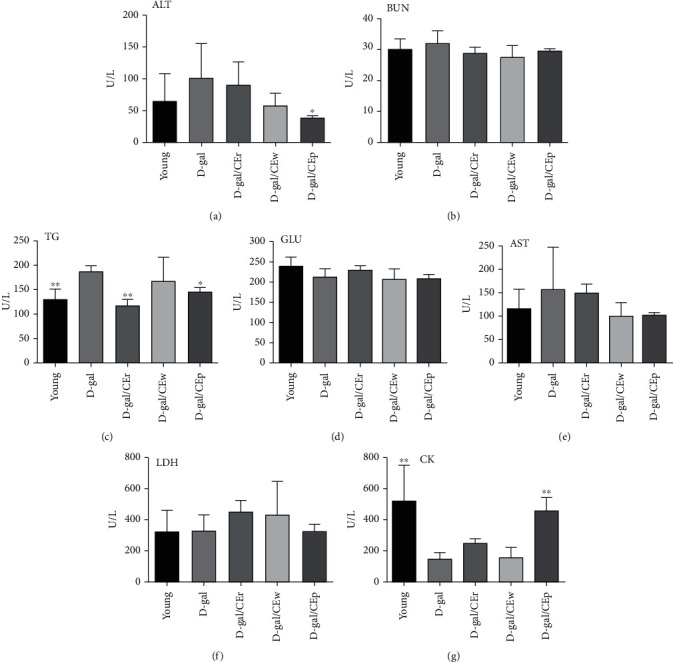
Effects of different parts of CEs on D-gal-induced serum biomarker concentration changes, including (a) ALT, (b) BUN, (c) TG, (d) GLU, (e) AST, (f) LDH, and (g) CK. Data are expressed as mean ± standard deviation (SD) in each group. ^∗^*P* < 0.05 or ^∗∗^*P* < 0.01 compared with the D-gal group.

Biochemical indices, including AST, BUN, LDH, and GLU, did not differ among groups (Figure 5). However, the TG levels were significantly lower by 16.1% in the D-gal/CEr group, when compared to the D-gal group. CK levels were significantly increased by 18.0% and by 31.7% in the D-gal/CEp and young groups, respectively, when compared to the D-gal group. Serum levels of ALT in the D-gal/CEp group were markedly lower than those in the D-gal group.

### 3.6. Effect of the Supplementation Using Different CE Parts on Antioxidative Enzyme Activity at the End of the Experiment

**Figure 6:**
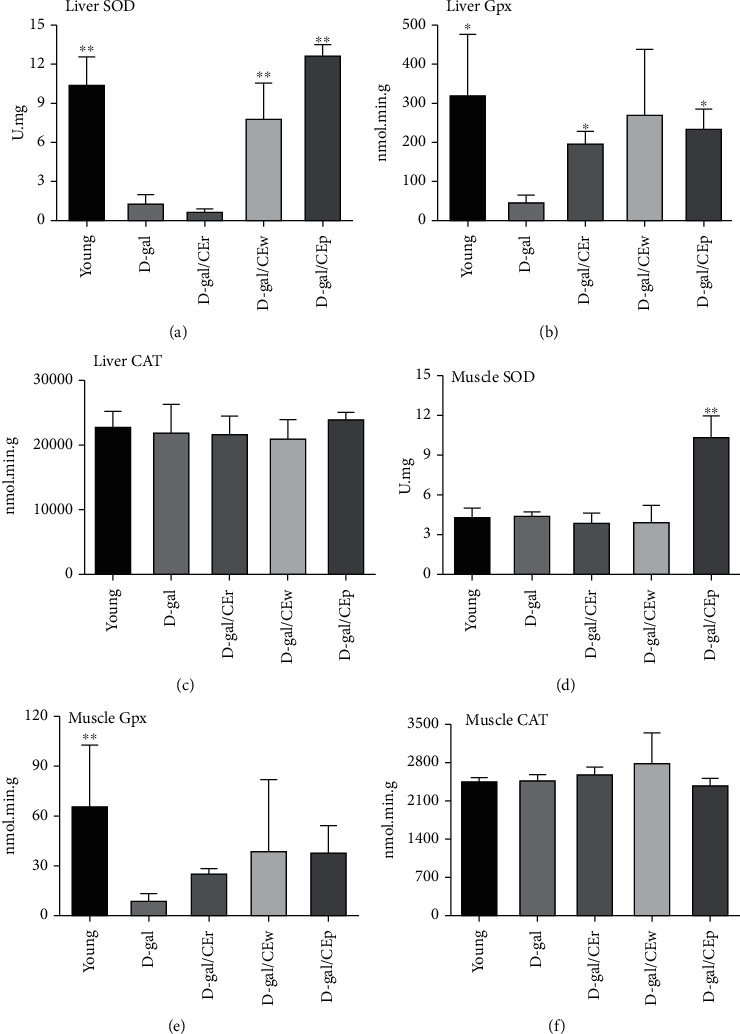
Effect of different parts of CEs supplementation on (a) liver SOD, (b) liver Gpx, (c) liver CAT, (d) muscle SOD, (e) muscle Gpx, and (f) muscle CAT, compared with D-gal group. Data are expressed as mean ± standard deviation (SD). ^∗^*P* < 0.05 or ^∗∗^*P* < 0.01 compared with the D-gal group.

As shown in Figure 6, the D-gal group showed a significant decrease in SOD and GPx in the liver, compared with the young group. There was a marked upregulation of the hepatic levels of SOD in the D-gal/CEp and D-gal/CEw groups and elevated levels of GPx in the D-gal/CEp and D-gal/CEr groups, compared with the D-gal group. In the muscles, no significant change was found in the level of GPx or CAT, while the level of SOD was significantly elevated in the D-gal/CEp group, compared with that in the D-gal group. Supplementation using different CE parts did not show an obvious effect on the level of CAT in each group, both in the liver and muscles.

### 3.7. Effect of Supplementation Using Different CE Parts on the Histological Examinations at the End of the Experiment

**Figure 7:**
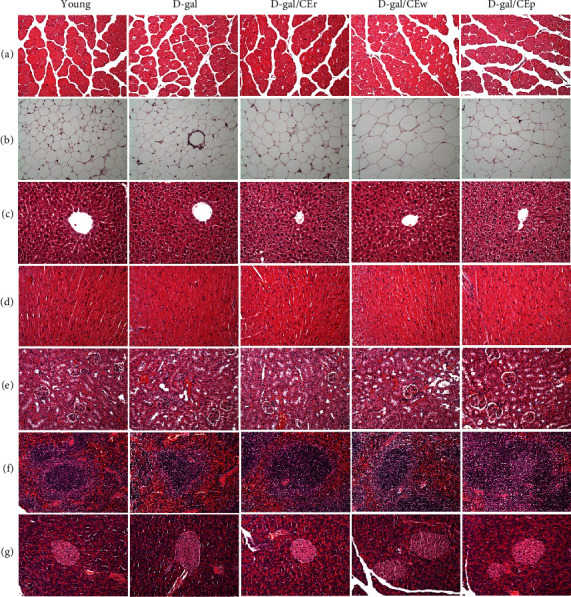
Effect of different parts of CEs on histological changes in (a) skeletal muscle, (b) fat tissue, (c) liver, (d) heart, (e) kidney, (f) spleen, and (g) pancreas tissues in the D-gal-induced aging model. Specimens were photographed using a light microscope (H&E stain, magnification: ×200; scale bar, 40 *μ*m).

The muscles, liver, heart, kidneys, epididymal fat pad, pancreas, and spleen of the mice of each group were studied via histological examination (Figure 7). The hepatocytes in the D-gal group contained a lower amount of cloud-like glycogen-containing cytoplasm, being slightly eosinophilic and having nuclear anisocytosis. However, the enrichment of glycogen and a uniform nucleus size were found in the livers of mice in the young and D-gal/CEp groups. In the muscle, a moderate grading of cytoplasmic vacuolation and fragmented myofibers was observed in the D-gal and D-gal/CEw groups. In contrast, these histologic changes were not seen in the young and D-gal/CEp groups; only slight lesions were observed in the D-gal/CEr group. In gonadal (epididymal) fat tissues, a slightly increased adipocyte size was found in the D-gal, D-gal/CEr, and D-gal/CEp groups, compared to the young group. Significantly larger adipocytes were seen in the D-gal/CEw group, compared to the other groups. Furthermore, crown-like structures (CLS), representing an accumulation of macrophages around dead adipocytes, were observed in the D-gal group. We did not observe these structures in the D-gal/CEp and young groups. No morphologic changes were observed in the heart, kidneys, and spleen of mice in each group.

### 3.8. Effect of Supplementation Using Different CE Parts on HO-1 and NRF-2 Expression Using Immunohistochemistry Staining

**Figure 8:**
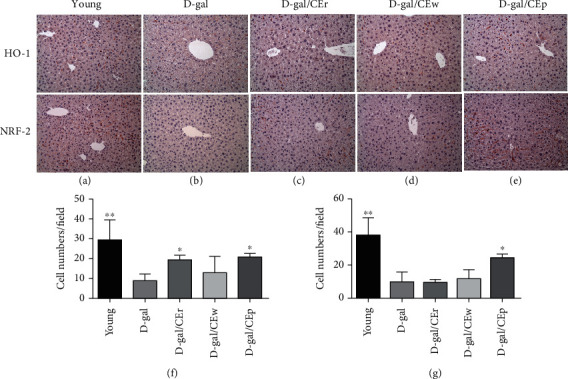
Immunohistochemistry staining for HO-1 and NRF-2 in liver sections. (a) Young, (b) D-gal, (c) D-gal/CEr, (d) D-gal/CEw, (e) D-gal/CEp, (f) HO-1 positive cells counting, and (g) NRF-2 positive cells counting. Magnification ×200. ^∗^*P* < 0.05 or ^∗∗^*P* < 0.01 compared with the D-gal group.

The HO-1 and NRF-2 positive signal cells in the liver tissue were analyzed by immunohistochemistry staining. A large number of HO-1 and NRF-2-positive signal cells were found in the D-gal/CEp group, compared with the D-gal group (Figure 8). The number of HO-1 positive signals cells was also markedly higher in the D-gal/CEr group. In contrast to the D-gal/CEp and young groups, the number of HO-1 and NRF-2 positive cells was decreased in the liver tissue of the D-gal group. A few NRF-2-positive cells were observed in the D-gal/CEr and D-gal/CEw groups.

## 4. Discussion

The present study is aimed at clarifying the antiaging and antifatigue effects of different parts of CE in a D-gal-induced aging mouse model. The chronic D-gal treatment successfully induced aging in the D-gal group, which was clearly different from the young group regarding by physiological fatigue test, histological sections, and biochemical indexes. Interestingly, the feeding with CEp could substantially ameliorate the D-gal caused aging process than in CEr or CEw group. The learning and memory impairments was improve seeming in CEr feeding mice. Moreover, the CEp have a good antioxidant activity, according to the hepatic SOD and GPx assays. Finally, histological assessment and IHC staining showed that CEp treatment could attenuate D-gal-induced skeletal muscle atrophy, hepatic glycogen decreases, and remarkably enhance NRF2 and HO-1 positive signals in the liver. These findings suggest that the CEp might be a major potential antiaging effects in the 10 days CE.

The muscles, fat tissue, and liver are important organs for energy regulation and storage. However, their functions gradually decline due to age-associated structural changes. Consistent with previous studies, middle-aged animals had increased body mass and fat tissue, but had a decreased muscle weight, compared to young mice [11]. In the present study, a decreased muscle weight (atrophy) and a higher lipid storage were found in the white fat tissue in the D-gal group. According to the weight of the organs and to the histologic evaluation results, in the D-gal/CEp group, but not in the D-gal/CEr and D-gal/CEw groups, these values and observations might be maintained. Skeletal muscles play important roles in extending a healthy life expectancy. Increases in muscle mass and strength are considered to be some of the major causes of health in older people. We suggest that supplementation with CEp might have similar ability as whey protein, leucine, and branched-chain amino acids, ameliorating muscle atrophy during aging [22].

Previous studies have shown that D-gal-induced aging in mice was caused by a poor intensity of the physiological challenges [6]. In the present study, a poor endurance, reduced physical performance, slow gait speed, and impaired mobility represent relevant aging in the D-gal group mice. However, a significantly improved motor coordination (rotarod), locomotion (OFT), muscle strength (forelimb and swim), and neuromuscular strength (hang test) were found in the D-gal/CEp group. The preadministration of CEp to the lesioned animals significantly modulates these behavioral changes.

During prolonged aerobic activity, depletion of muscle glycogen often occurs and is correlated with the inability to maintain the muscle contraction force [18]. On the other hand, muscle atrophy and muscle mass loss are usually seen in aging animals, compared to young animals. In our previous studies, glycogen storage in skeletal muscles exhibited a dramatic reduction in the swimming-induced fatigue model, after fatigue induction [23]. In present study, higher levels of glycogen content in the D-gal/CEp and D-gal/CEr groups, compared to the D-gal group, indicate a higher glycogen store in the muscles via CEp and CEr supplementation. Only the D-gal/CEp group had increases in the glycogen content in the liver. These finding may directly increase exercise performance and reduce physical fatigue in mice that receive CEp supplementation, ameliorating the D-gal-induced aging process. However, further studies are needed to confirm this hypothesis.

Our results showed that less glycogen was present in the D-gal group than in the young group. These data agree with those of other rodent experiments [10]. Age-related inhibition of hepatocyte proliferation has previously been reported to be related to a decrease in glycogen synthase-3*β* expression. These pivotal roles regulate hepatocyte proliferation. Similar to previous reports, a significant increase in the fat tissue weight was observed in the D-gal group, compared to the young group, which may be a result of adipocyte hypertrophy. Interestingly, a marked hypertrophy of adipocytes and an increased fat tissue weight were observed in the D-gal/CEw group, compared to the other groups. This might be due to the high intake of carbohydrates in the CEw containing. We also confirmed that the D-gal/CEp and D-gal/CEr groups do not induce adipocyte hypertrophy. In previous study, a higher percentage of atrophic muscle fibers and degeneration changes was found in old mice, compared to young mice [24]. The D-gal-induced muscle morphologic changes were similar to those of our previous study. These changes might cause aging-related disorder such as reduction in skeletal muscle mass (sarcopenia), which is generally associated with muscle weakness. These aging-related morphologic changes were improved via CEp feeding. Therefore, we suggest that the CEp inhibition of muscle atrophy/degeneration might be via antioxidant enzyme activity and rich nutrients contain. However, the detailed molecular mechanism still needs further experimental proof.

It is widely accepted that the redox equilibrium is involved in the aging process. As part of the antioxidant defense systems, a group of enzymes, including CAT, SOD, and GPx, function as superoxide anions and hydrogen peroxide scavengers to prevent ROS-induced damage [4, 25]. The present results indicate that CEp markedly diminished the oxidative stress in aged mice by increasing the activities of SOD in the liver and muscles, and of GPx in the liver. A markedly increased GPx was found in the D-gal/CEr group. These findings suggest that different parts of the CE might have partial effects on antioxidative stress in the aging process.

Recently, the NRF-2 and HO-1 was found to be an important regulator of body resistance to oxidative stress [26]. It also prevents a number of aging-related diseases such as hypertension, sarcopenia, and Alzheimer's disease [27, 28]. The NRF-2 could be accompanied with HO-1 to regulate the expression of many antioxidant and phase II detoxifying enzyme genes, such as NADP(H) and quinine oxidoreductase-1 (NQO1). In this study, the large number of HO-1 positive cells were found in the D-gal/CEp and D-gal/CEr groups, and NRF-2-positive signal cells were found in the D-gal/CEp group. These finding were partially consistent with marked upregulation of the hepatic levels of antioxidase enzymes including SOD in the D-gal/CEp and D-gal/CEw groups, and GPx in the D-gal/CEp and D-gal/CEr groups. These results might indicate that the parts of CE (especially CEp) activation of the NRF-2/HO-1 pathway have a protective effect against D-gal-induced aging via antioxidative mechanisms.

Taking together, we showed the better antiaging and antifatigue performance of the mice in the D-gal/Cep, compared to those in the D-gal/CEr and D-gal/CEw groups, in a long-term ingestion D-gal aging model. In addition, we suggest that CEp might through HO-1/NRF-2 signal pathway to induce and activate SOD and GPx and maintain the glycogen levels to ameliorate D-gal-induced aging. These data might provide the reference basis of antiaging, antifatigue functional evidence of different parts of chick embryo egg-related products.

## Figures and Tables

**Table 1:** Nutritional facts.

	CEp	CEr	CEw
Calories (kcal/100 g)	158.01	30.72	250.78
Nutritional facts (%)			
Moisture	61.42	92.90	52.31
Ash	0.74	0.92	0.82
Crude fat	1.33	1.20	12.66
Crude protein	31.96	4.40	7.56
Total carbohydrates	4.55	0.58	26.65

## Data Availability

All the data used to support the finding of the present study are presented in the article. The original data of the study are available from the corresponding author upon request.

## Supplementary Materials

Supplementary Materials Figure S1: different parts of CE supplementation improve D-gal-induced aging model. The body weight recorded at different time points for the beginning, 1 month (mo), 2 mo, 3 mo, and end of experiment.
