# Supplementary material for: Different Parts of the Chicken Embryo Egg Improve D-Galactose-Induced Aging in a Mice Model
Source: Biomed Res Int. 2021 Apr 27;2021:6654683. doi: 10.1155/2021/6654683 (PMC8099523; doi:10.1155/2021/6654683)
Supplement: Supplementary Materials — Figure S1: different parts of CE supplementation improve D-gal-induced aging model. The body weight recorded at different time points for the beginning, 1 month (mo), 2 mo, 3 mo, and end of experiment. [file 6654683.f1.pptx]

## Slide 1
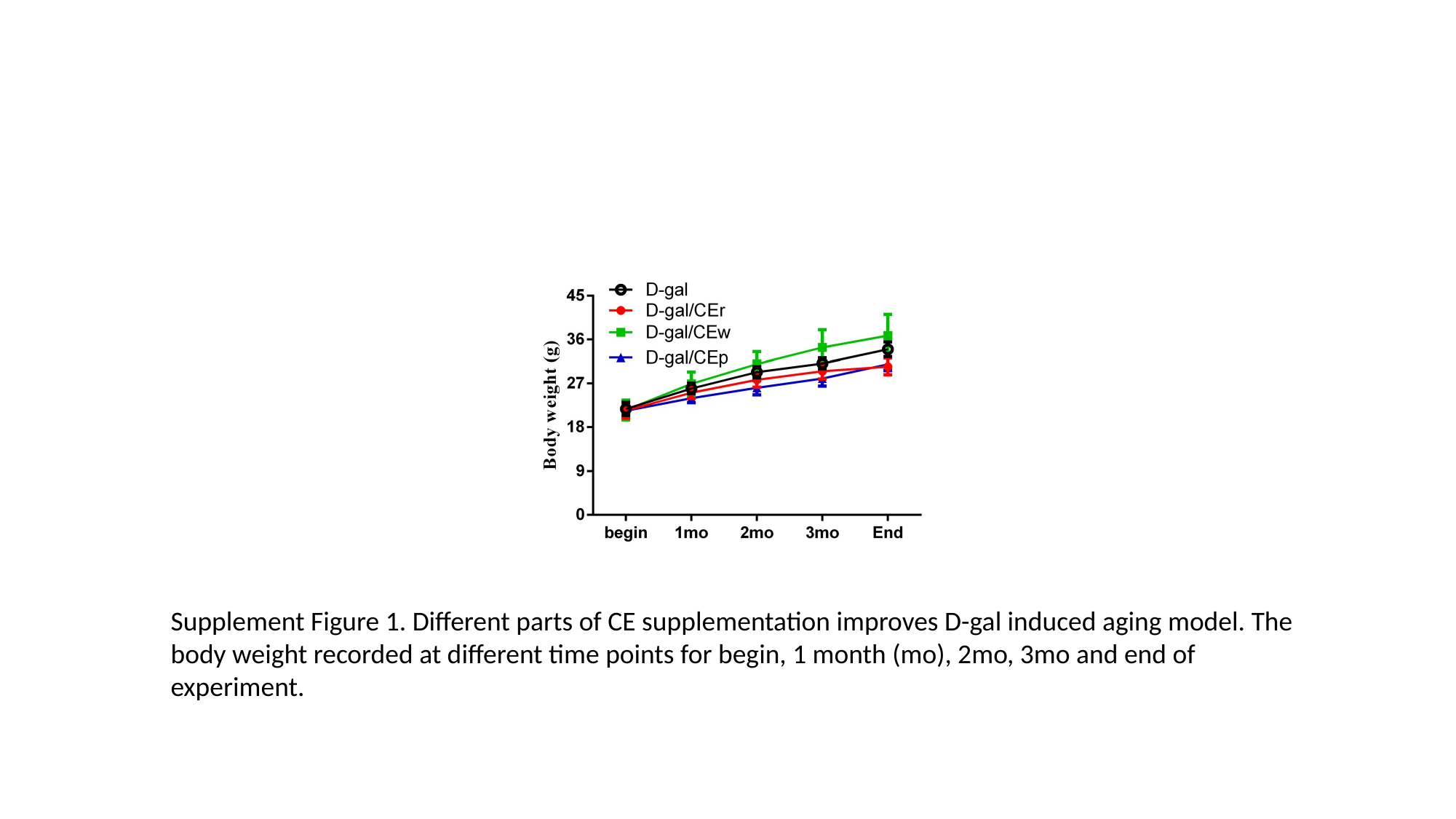

Supplement Figure 1. Different parts of CE supplementation improves D-gal induced aging model. The body weight recorded at different time points for begin, 1 month (mo), 2mo, 3mo and end of experiment.
